# STAT3 Differentially Regulates TLR4-Mediated Inflammatory Responses in Early or Late Phases

**DOI:** 10.3390/ijms21207675

**Published:** 2020-10-16

**Authors:** Akash Ahuja, Eunji Kim, Gi-Ho Sung, Jae Youl Cho

**Affiliations:** 1 Department of Integrative Biotechnology, and Biomedical Institute for Convergence at SKKU (BICS), Sungkyunkwan University, Suwon 16419, Korea; akash.ahuja1988@gmail.com (A.A.); im144069@gmail.com (E.K.); 2 Department of Microbiology, Biomedical Institute of Mycological Resource, International St. Mary’s Hospital and College of Medicine, Catholic Kwandong University, Simgokro, 100 Gil, 7, Seo-gu, Incheon 22711, Korea

**Keywords:** inflammation, Akt, COX-2, iNOS, TLR4, STAT3, NF-κB, macrophages

## Abstract

Toll-like receptor 4 (TLR4) signaling is an important therapeutic target to manage lipopolysaccharide (LPS)-induced inflammation. The transcription factor signal transducer and activator of transcription 3 (STAT3) has been identified as an important regulator of various immune-related diseases and has generated interest as a therapeutic target. Here, we investigated the time-dependent roles of STAT3 in LPS-stimulated RAW264.7 macrophages. STAT3 inhibition induced expression of the pro-inflammatory genes *iNOS* and *COX-2* at early time points. STAT3 depletion resulted in regulation of nuclear translocation of nuclear factor (NF)-κB subunits p50 and p65 and IκBα/Akt/PI3K signaling. Moreover, we found that one Src family kinase, Lyn kinase, was phosphorylated in STAT3 knockout macrophages. In addition to using pharmacological inhibition of NF-κB, we found out that STAT3KO activation of NF-κB subunit p50 and p65 and expression of iNOS was significantly inhibited; furthermore, Akt tyrosine kinase inhibitors also inhibited iNOS and COX-2 gene expression during early time points of LPS stimulation, demonstrating an NF-κB- Akt-dependent mechanism. On the other hand, *iNOS* expression was downregulated after prolonged treatment with LPS. Activation of NF-κB signaling was also suppressed, and consequently, nitric oxide (NO) production and cell invasion were repressed. Overall, our data indicate that STAT3 differentially regulates early- and late-phase TLR4-mediated inflammatory responses.

## 1. Introduction

The innate immune response, including inflammatory cytokine production, immune cell recruitment, and phagocytosis by macrophages and neutrophils, plays critical role in host defense during the early stages of infection [1]. Inflammatory responses are stimulated by activation of Toll-like receptors (TLRs), which are critical components of the innate immune response based on their ability to recognize pathogen-associated molecular patterns (PAMPs) [2]. Lipopolysaccharide (LPS) is a TLR4 ligand and forms complexes with TLR4 to induce specific signaling pathways involving various mediators, such as MyD88 and TRAF6, which direct NF-κB/Rel subunits into the nucleus and regulate inflammatory gene expression [3]. Initiating innate immune responses against pathogens is also linked to transmitting the signals for antigen specific adaptive immune responses [4].

Primary phagocytes, also known as macrophages, contribute significantly to host defense and regulation of inflammation [5]. Macrophages are known to transform into different phenotypes of classically activated M1 and alternatively activated M2 dependent on stimuli. For example, stimulation with bacterial lipopolysaccharide (LPS), a known compound present on the cellular wall of Gram-negative bacteria, produces an M1 subtype [6,7] to induce an inflammatory response [8]. Activated macrophages produce nitric oxide (NO) and secrete pro-inflammatory cytokines like interleukin (IL)-1β, IL-6, and TNF-α, which activate a plethora of cell signaling pathways including Janus kinase 2/signal transducers and activators of transcription 3 (JAK/STAT3), mitogen-activated protein kinase (MAPK)/activator protein (AP)-1, and IκBα kinase (IKK)/nuclear factor-κB (NF-κB) [9,10,11,12]. STAT3 signaling has been reported to play an important role in macrophage polarization to M2 phenotype [13]. The JAK/STAT3 signaling cascade has also been designated as an essential signaling pathway in mediating immune response [14]. RAW264.7 cells are a good model cell line to study the functional role of M1 and M2 macrophages as well as to screen anti-inflammatory and immunostimulatory drugs [15,16,17].

Gene expression and transcriptomics analyses suggest that the signal transducer and activator of transcription (STAT) family of proteins is activated in response to infection [18,19]. In particular, STAT3 represents an important transcription factor activated by a variety of cytokines and growth factors [20], which translocate to the nucleus and regulate genes responsible for proliferation, migration, inflammation, and apoptosis depending on the cell type [21,22]. Cytokines such as IL-6 and IL-10 are associated with STAT3 activation and regulate multiple cellular functions [23]. STAT3 is also known to play an important role in autoimmune diseases, including inflammatory bowel disease (IBD) and *Mycobacterium tuberculosis* infection [24]. Moreover, it is well known that STAT3 transcription factors are involved in multiple adaptive immune responses [25,26].

STAT3 deletion in hematopoietic cells is known to lead to the development of colitis because of negative regulation of STAT3 in colon inflammatory responses [27]; on the other hand, STAT3 deletion in T-cells appears to provide protection from a variety of autoimmune diseases [28], indicating independent roles for STAT3 in different cell types. STAT3 deletion is also known to reduce tumor growth and prolongs survival in mouse models [29]. STAT3 inhibition has also shown preventive effects in controlling lung inflammation and Th2 cell differentiation in mouse models with asthma [30]. Moreover, plant-derived natural compounds have also been observed to be effective in inflammatory and cancerous conations by inhibiting STAT3 and nuclear factor (NF)- κB transcription factors and various pro-inflammatory cytokines [31,32]. Small molecule inhibitors of JAK2/STAT3 signaling like FLLL32 reduced the growth of established neurofibromas in mouse models [33]. Numerous pharmacological inhibitors have been developed for targeting NF-κB and STAT3 to treat various cancers and inflammatory disorders [34]. Studies have also showed that genetic ablation of STAT3 in gp130^F/F^ showed hypersensitivity and increase in IL6 levels in response to LPS [35].

Macrophages display different gene expression and protein activity according to duration of LPS exposure, and STAT3 plays an active role in the inflammatory process [36,37,38]. We wished to investigate the intracellular inflammatory signaling of STAT3 at several time points during LPS exposure in vitro. To investigate the role of STAT3 within LPS-exposed macrophages, we depleted STAT3 in RAW264.7 macrophage-like cells using the CRISPR-Cas9 system. We further elucidated the whole inflammatory signaling pathways which are known to be regulated in macrophages upon LPS stimulation in early as well as late time points; our analysis revealed translocation of NF-κB subunit p65 in STAT3 knockout (STAT3KO) cells starting at 5 min after LPS treatment and regulated upstream Lyn tyrosine kinase. In addition, we found that STAT3KO induced mRNA expression of *iNOS* and *COX-2* in an early time point. We further demonstrated these effects in the late phase of LPS stimulation by which iNOS gene expression and nitric oxide production were decreased. Last, we show that STAT3KO decreased the cell invasion under LPS-stimulated conditions. Taken together, our study demonstrates the underlining molecular mechanism targeting STAT3 activity in macrophages.

## 2. Results

### 2.1. Establishment of STAT3 Knockout RAW264.7 Macrophage Cell Line

**Figure 1:**
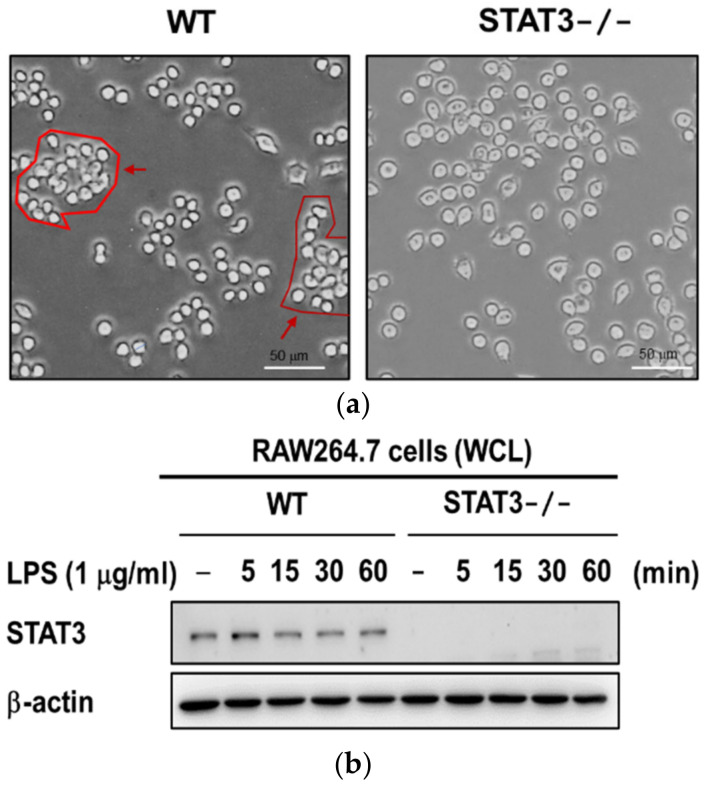
Confirmation of STAT3 knockout in STAT3KO macrophages. (**a**) Cell morphology in macrophages from WT and STAT3KO cells. Normal macrophages to the left showed grouped morphology while STAT3-deficient cells on the right exhibited disk-like morphology and did not form groups; images were documented at 40× magnification and 50 μm scale bar. (**b**) Western blot analyses to confirm the levels of STAT3 in wild type and STAT^-/-^ RAW264.7. Wild type and STAT3^-/-^ cells were stimulated with LPS for 0, 5, 15, 30, and 60 min. Total cell lysates were subjected to immunoblotting using antibodies to STAT3 and β-actin. WCL: whole cell lysates.

Phosphorylation and activation of JAK/STAT3 has been described as a chronic phenomenon which is evidenced by STAT3 activation [39,40]. We generated a STAT3 knockout (STAT3KO) RAW264.7 macrophage cell line by using a CRISPR-CAS9 gene editing tool. We first examined the phenotype of STAT3KO and wild type (WT) macrophages to investigate whether STAT3 depletion affected cell structure: images of STAT3KO macrophages showed that morphology of these cells are very similar to those of wild type cells (Figure 1a). We then confirmed the efficiency of the STAT3 knockout procedure using immunoblotting against STAT3 antibody. As shown in Figure 1b, Western blot analyses confirmed STAT3 was stably knocked out in RAW264.7 cells as compared to WT cells. Taken together, these results demonstrated that the CRISPR-CAS9 tool successfully stably targeted the STAT3 gene. In this study, we investigated the role of STAT3 in LPS-induced inflammatory responses using STAT3KO macrophages.

### 2.2. STAT3 Deletion in Macrophages Leads to Enhanced Lyn/AKT/NF-κB-Dependent Inflammatory Signaling Pathways in Early Stages of Inflammation

**Figure 2:**
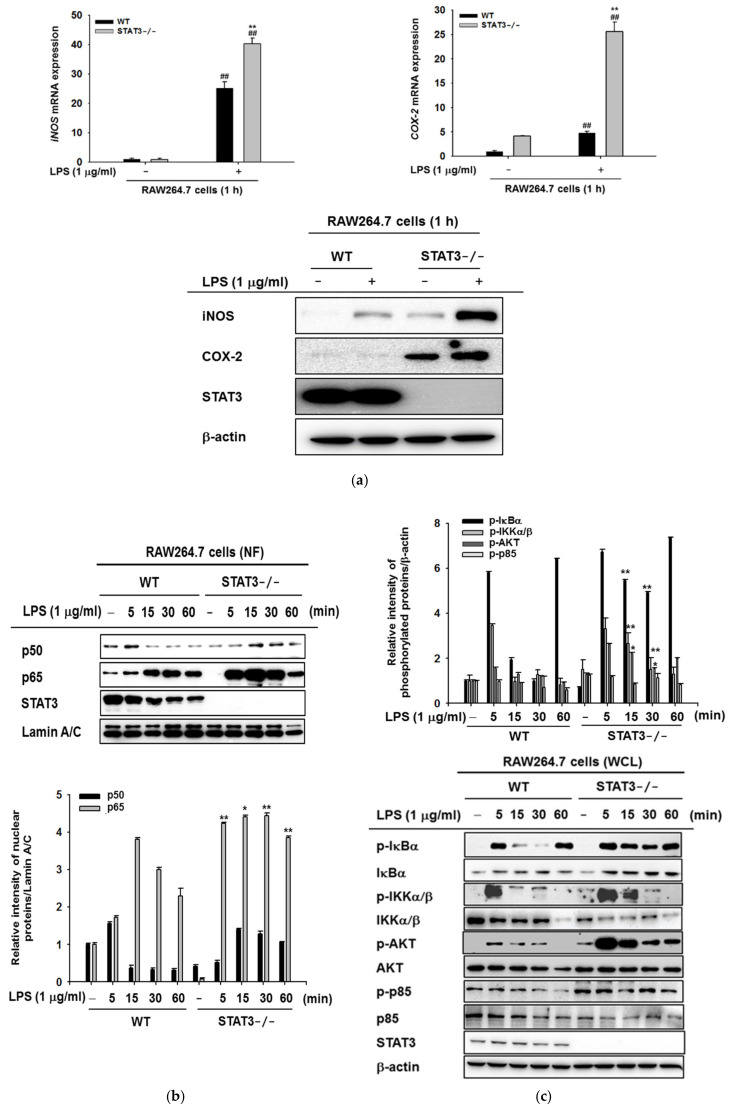

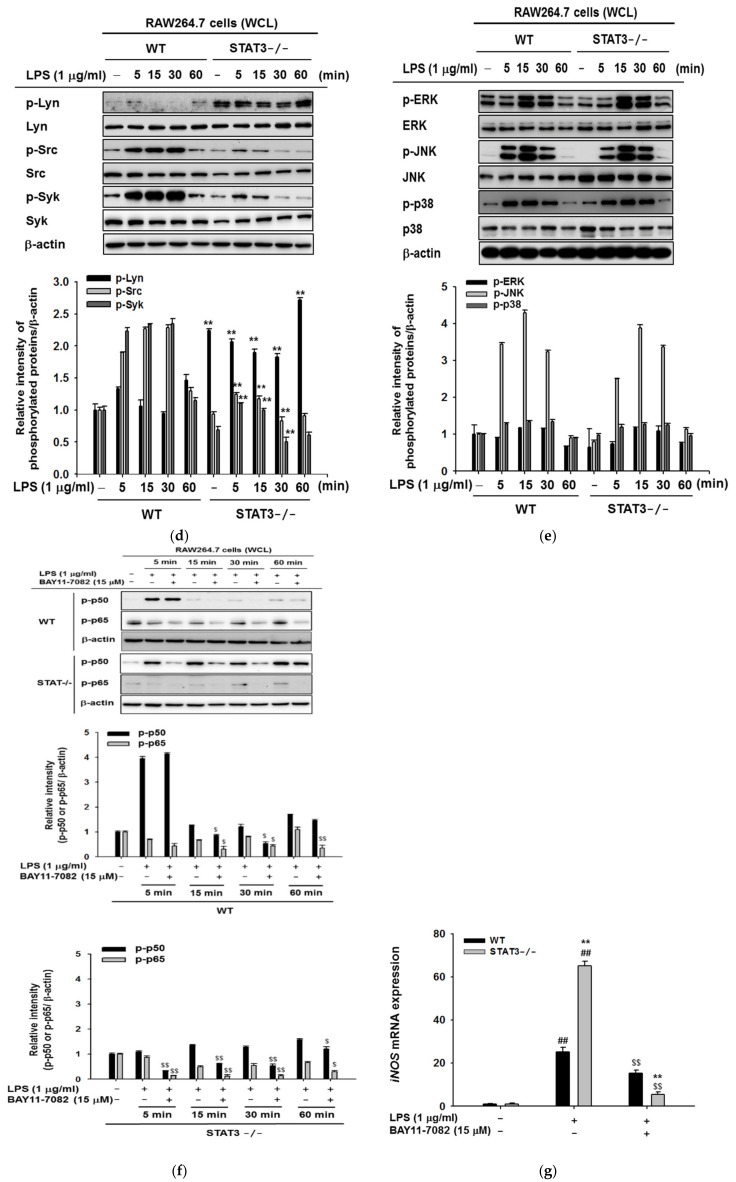

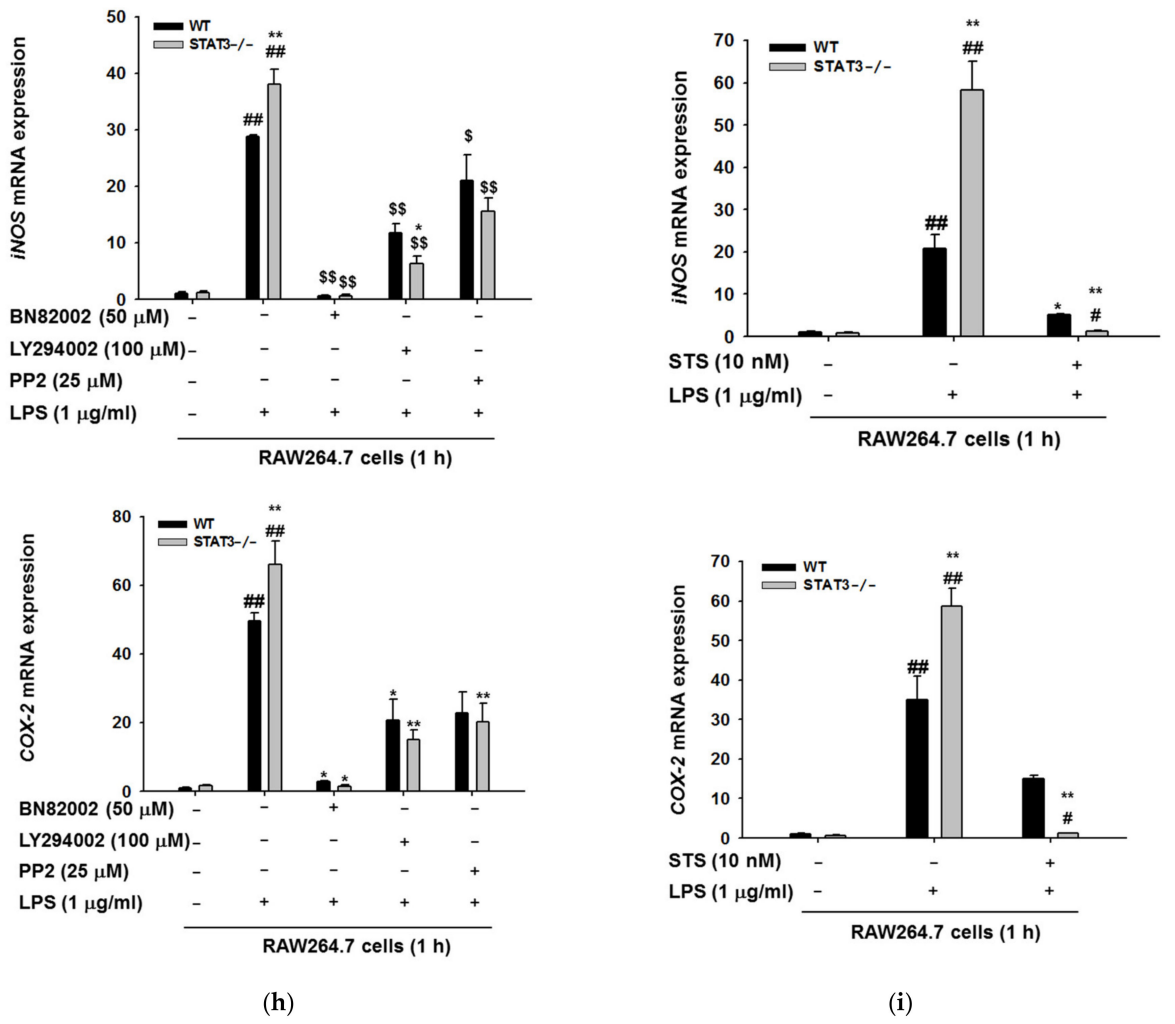
Early effects of LPS on *iNOS* and *COX-2* and NF-κB-dependent inflammatory signaling pathways in WT and STAT3KO macrophages. (**a**) The mRNA expression of *iNOS*, *COX-2*, and their protein levels were measured after 60 min incubation with LPS as assessed by qRT-PCR and immunoblotting blotting analyses. Values represent average concentrations of the indicated cytokines. (**b**) Wild type and STAT3^-/-^ RAW264.7 cells were incubated with LPS (1 μg/mL for 0–60 min) and the nuclear fractions of p50 and p65 were examined using immunoblotting blotting analyses with lamin A/C used as an internal control. (**c**) Wild type and STAT3^-/-^ RAW264.7 cells were stimulated with 1 μg/mL LPS for the indicated period of time. Whole-cell lysates were then subjected to immunoblotting to identify phosphorylation and total forms of inhibitor of kappa B (IκBα,), IκBα kinase α/β (IKKα/β), and protein kinase B (Akt), with β-actin as an internal control. (**d**) Immunoblotting was performed in wild type and STAT3^-/-^ RAW264.7 cells were left untreated or incubated with LPS for the indicated period. Phosphorylated and total forms of Lyn, Syk, Src, and p85 were examined. β-actin was used as the internal control. (**e**) The MAPK signaling pathway activation (as represented by ERK, p38, and JNK) was also assessed after LPS (1 μg/mL) stimulation in wild type and STAT3^-/-^ RAW264.7 macrophages, using β-actin as an internal control. (**f**) WT and STAT3KO cells were treated with BAY11-7082 (15 μM) for 30 min prior to LPS (1 μg/mL) treatment for 5–60 min. Whole cell proteins were isolated and immunoblotted with indicated antibodies to p-p65 and p-p50. (**g**) WT and STAT3KO cells were treated with BAY11-7082 (15 μM) for 30 min prior to LPS (1 mg/mL) treatment for 6 h. Isolation of RNA, generation of cDNA, and quantitative RT-PCR were performed for *iNOS* determination. (**h**,**i**) RAW264.7 and STAT3KO macrophages were pre-treated with or without 50 μM of BN82002, 100 μM of LY294002, 25 μM of PP2, or 10 nM of staurosporine (STS) for 30 min, followed by LPS (1 μg/mL) stimulation. qRT-PCR was used to evaluate iNOS (upper panels) and COX-2 (lower panels) mRNA gene expression. Data represent mean ± SD, replicates of three independent experiments. Relative intensity is defined as the intensity of the target protein normalized to β-actin. Values are presented as the mean ± standard deviation. ^##^ *p* < 0.01 compared to normal group in the same cell type, ^$^ *p* < 0.05 and ^$$^ *p* < 0.01 compared to LPS alone, and * *p* < 0.05 and ** *p* < 0.01 compared to the same condition of wild type. NF: nuclear fractions. WCL: whole cell lysates.

To identify potential genes regulated by STAT3 in LPS-stimulated macrophages, we analyzed the expression of genes associated with inflammatory responses in WT and STAT3KO macrophages stimulated with LPS for 1 h. The levels of pro-inflammatory genes were detected using real-time PCR and immunoblotting analysis at the mRNA and protein levels. The analysis of mRNA (left and right panels) and protein levels (bottom panel) of *iNOS* and *COX-2* revealed that *iNOS* (left panel) and *COX-2* (right panel) were highly increased in STAT3KO macrophages compared to WT macrophages in the absence or presence of LPS (Figure 2a). The results imply that STAT3 negatively regulated the pro-inflammatory genes (*iNOS* and *COX-2*) in macrophages that were briefly exposed to LPS.

Macrophages promote inflammation by releasing pro-inflammatory cytokines and chemokines, such as IL-6, TNF-α, and IL-1β [41]. In addition, transcription of *iNOS* and COX-2, which are related to inflammation, cancer, and other inflammatory disorders, is stimulated by NF-κB [42,43]. Moreover, activation of NF-κB involves nuclear translocation of the NF-κB subunits p50 and p65; therefore, we first examined nuclear p50 and p65 content in nuclear lysates from LPS-treated WT and STAT3KO macrophages in a time-dependent manner. In our experiments, p65 was found to be translocated into the nucleus starting as early as five minutes following LPS treatment in STAT3KO groups, whereas p50 was observed minimal or no different between WT and STAT3KO under LPS stimulation (Figure 2b). To determine whether the upstream kinetics of NF-κB signaling was affected by loss of STAT3 function, analysis of phospho-IκBα and IKKα/β was performed by immunoblotting. As shown in Figure 2c, increase in IκBα was observed at 5 and 60 min in WT post LPS exposure, with significant decreases at 15 and 30 min, whereas the phosphorylation level of IκBα consistently increased from 5 to 60 min in STAT3KO macrophages post LPS exposure, suggesting persistent phosphorylation of ΙκBα in STATKO macrophages. The kinetic profile of IKKα/β phosphorylation was increased at 5 min in WT and STAT3KO macrophages, and the patterns were similar. These results imply a possibility that STAT3 can participate in controlling earlier translocation of p65 into the nucleus leading to NF-κB activation under LPS stimulation conditions.

Akt is critically involved in regulation of programmed cell death; additionally, NF-κB activation is reported to require Akt [44,45]. Interestingly, we found that LPS treatment induced greater phosphorylation of Akt in STAT3KO macrophages upon LPS stimulation in comparison to WT macrophages (Figure 2c). However, phosphorylation of p85 produced no significant difference between WT and STAT3KO macrophages (Figure 2c). Altogether, our results suggest that involvement of STAT3 in regulation of *iNOS* and *COX-2* gene expression at early time points contributes to enhanced activation of NF-κB and Akt in STAT3KO macrophages as reported by others [46].

The above results led us to investigate the upstream mechanism regulated by STAT3 under LPS stimulation; we next examined the upstream kinase proteins involved in activation of inflammatory signaling pathways at early time points. Given that Src, Syk, and Lyn kinases are involved in NF-κB activation in various conditions, such as TNF, hydrogen peroxide, or TLR activation [47,48,49,50,51], we examined the activated level of these three kinases (Figure 2d). Of note, Lyn is localized to the membrane [52], and Src and Lyn are the sole members of Src family tyrosine kinases [53]. Previous reports suggested that overexpression of Lyn tyrosine kinase increases liver injury [54]; moreover, knockdown of Lyn impaired NF-κB pathway activation [55]. With the aim to evaluate the role of Lyn kinase in our system, we performed immunoblotting analysis and determined time-dependent phosphorylation pattern of Lyn. Interestingly, phosphorylation level of Lyn in STAT3KO cells was consistently increased in STAT3KO cells with or without LPS (Figure 2d). LPS treatment to STAT3KO cells also enhanced the phosphorylation of Lyn up to 34% at 60 min, than in wild type cells (Figure 2d), implying that STAT3KO Lyn could play an important role in activation of NF-κB signaling in early phase of inflammation. On the other hand, phosphorylated Src and Syk were increased in WT macrophages as compared to STAT3KO cells. These results implied that STAT3 regulates basal activity of Lyn by controlling its phosphorylation which can be upregulated by LPS at 1 h. Intriguingly, there were no significant differences in the phosphorylation of ERK, p38, and JNK (MAPK pathway) in WT and STAT3KO macrophages (Figure 2e). Collectively, these results suggested a putative functional role of STAT3 in NF-κB signaling through phosphorylation of the Lyn tyrosine kinase and its substrate p85, which can be boosted by TLR4 stimulation with LPS.

Results shown above indicate that blocking of STAT3 in RAW264.7 macrophages leads to activation of NF-κB signaling and increases in *iNOS* and *COX-2* gene expression in early time points of LPS stimulation. In order to verify the role of the NF-κB pathway, we used a pharmacological inhibitor, BAY11-7082 (BAY), of the NF-κB pathway, as reported previously [56]. WT and STAT3KO cells were pre-treated with BAY for 30 min followed by LPS (1 μg/mL) stimulation for indicated period (5–15 min). We checked the effect of BAY11-7082 on phosphorylation of NF-κB subunits, p50 and p65. As shown in Figure 2f, phosphorylation levels of p50 and p65 were increased in LPS-stimulated groups of both WT and STAT3KO macrophages, although some time points (15, 30, and 60 min) showed striking enhancement of p-p65 in WT and p-p50 in STAT3KO cells. Nonetheless, BAY11-7082 exposure significantly suppressed phosphorylation levels of both p65 and p50 in both WT and STAT3KO macrophages at 15 and 30 min (Figure 2f). To further test the role of NF-κB pathway in *iNOS* gene expression, we pretreated BAY for 30 min followed by LPS (1 μg/mL) for 6 h, and quantitative RT-PCR was performed to measure the level of *iNOS*. LPS treatment induced mRNA expression of *iNOS* in STAT3KO macrophages, which was inhibited by pre-treatment with BAY (Figure 2g). Moreover, WT macrophages also observed similar gene expression patterns; however, *iNOS* gene expression was observed to be significantly reduced in STAT3KO macrophage (Figure 2g), suggesting that NF-κB is necessary for *iNOS* gene expression in STAT3KO cells. Since STAT3KO macrophages showed higher levels of phosphorylated Lyn tyrosine kinase and Akt in response to LPS stimulation and significantly increased *iNOS* and *COX-2* expression in comparison with WT macrophages (Figure 2a,d), whether Akt and Lyn are directly involved in controlling the expression of *iNOS* and *COX-2* was examined. To do this, pharmacological inhibitors of Akt (BN82002 or LY294002) and Src/Lyn (PP2 and staurosporin) [57,58] were employed. As shown in Figure 2h,i, pretreatment with BN82002, LY294002, PP2, and staurosporin (STS) resulted in inhibition of LPS-induced *iNOS* and *COX-2* in both WT and STAT3KO macrophages within 1 h. These findings suggest that dual Lyn and AKT might play critical roles in upregulation of iNOS and COX-2 under STAT3KO conditions.

### 2.3. STAT3 Deletion in Macrophages Display Reduced Gene Expressions Profiles of iNOS and IL-10, and NF-κB Signaling in Macrophages at Later Stages of LPS Stimulation

**Figure 3:**
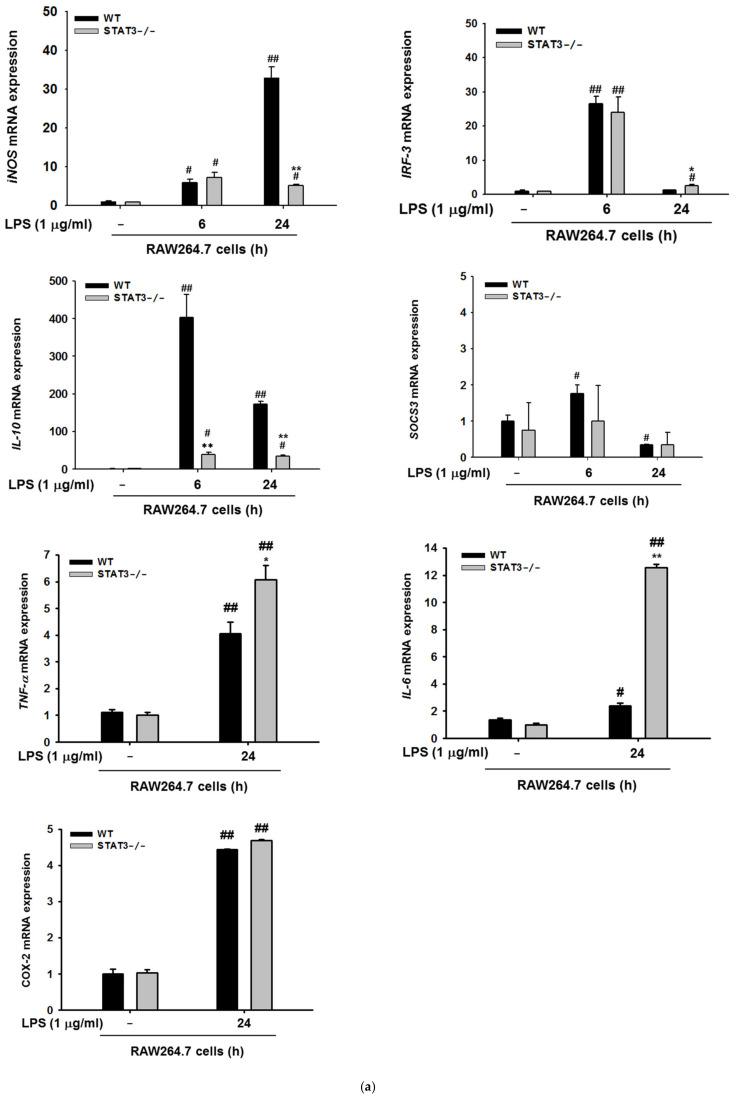

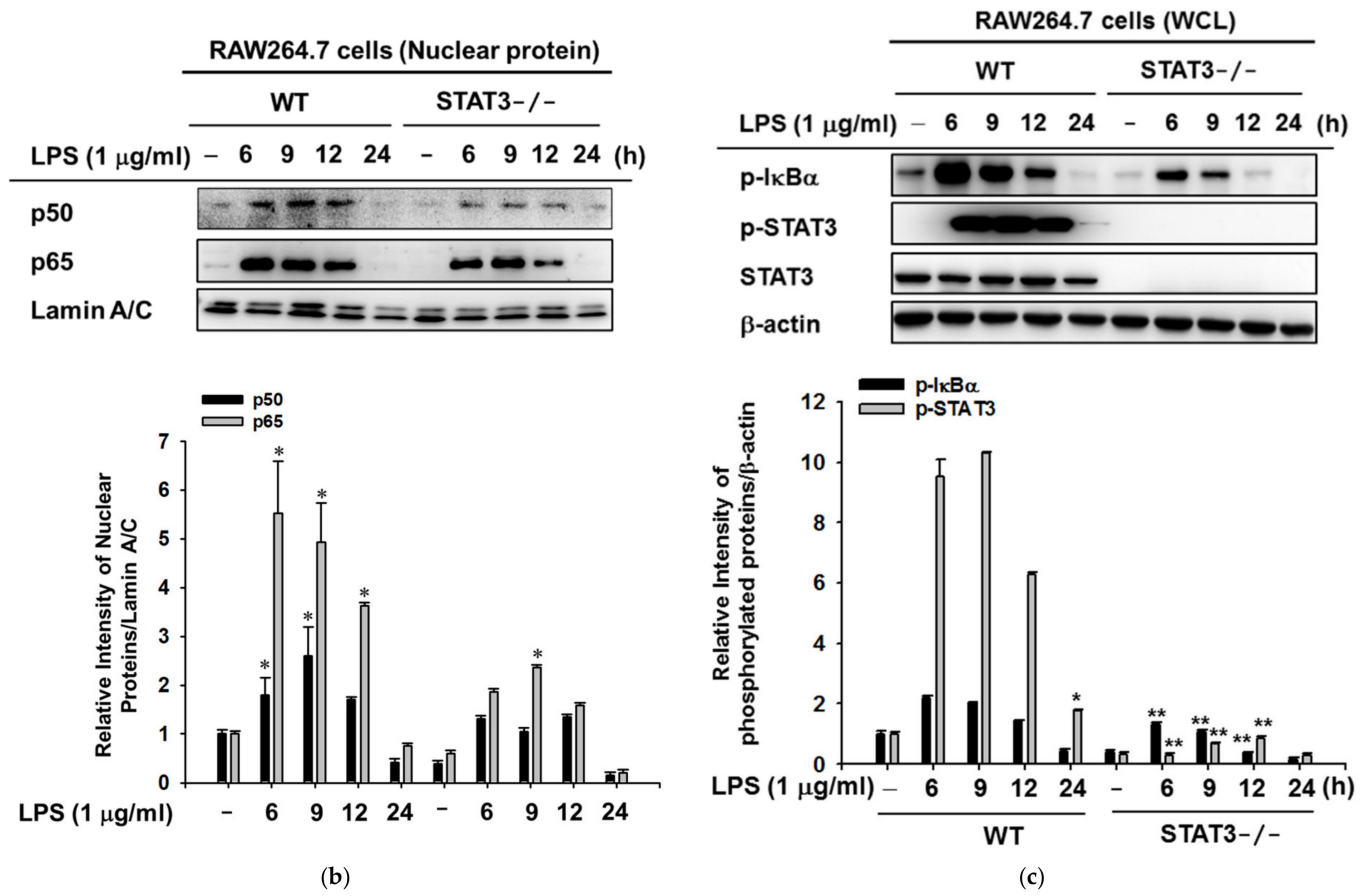
Effects of LPS on *iNOS* gene expression and NF-κB signaling at later time points. (**a**) mRNA expression of *iNOS*, *TNF-α*, *IL-6*, *COX-2*, *IL-10*, *IRF-3*, and *SOCS3* measured at 6 and 24 h after LPS treatment, in RAW264.7 wild type and STAT3^-/^ cells^-^. Values represents average concentrations of the indicated cytokines. Data are presented as the mean ± SD, for replicates of three independent experiments calculated using Student’s *t*-test (* *p* < 0.05, ** *p* < 0.01 as compared with the control (untreated) group). (**b**) Wild type and STAT3^-/-^ RAW264.7 cells were incubated with LPS (1 μg/mL for 0–24 h) and nuclear fractions of p50 and p65 were examined by immunoblotting analyses, using lamin A/C as an internal control. (**c**) Wild type and STAT3^-/-^ RAW264.7 cells were stimulated with 1 μg/mL LPS for the indicated period and whole cell lysate were then subjected to immunoblotting against IκBα and STAT3 with β-actin as an internal control. ^#^*p* < 0.05 and ^##^ *p* < 0.01 compared to normal group in the same cell type and * *p* < 0.05 and ** *p* < 0.01 compared to the same condition of wild type. NF: nuclear fractions. WCL: whole cell lysates.

To further determine the role of STAT3, we next studied the suppressive effect of STAT3 using macrophages lacking STAT3 and examined gene expression of NF-κB/STAT3-dependent *iNOS*, *TNF-α*, *IL-6*, *IL-10*, *SOCS3*, and *IRF-3* in later stages of LPS stimulation (0–24 h). Incubation with LPS resulted in time-dependent induction of *iNOS* gene expression in WT macrophages, whereas STAT3KO macrophages generated significantly less expression of *iNOS* at 24 h in response to LPS stimulation (Figure 2a). We next demonstrated the effect of STAT3 knockout on the mRNA expression of *IL-10*, *SOCS3*, and *IRF-3*. As shown in Figure 3a, the expression of IL-10 in WT macrophages was increased compared to their STAT3KO counter parts under LPS stimulation conditions, whereas the level of *COX-2*, *SOCS3*, and *IRF-3* were not significantly different between WT and STAT3KO groups upon LPS treatment. However, mRNA levels of TNF-α and IL-6 were strongly enhanced in STAT3KO cells (Figure 3a). We next tested the nuclear translocation of the NF-κB subunits p50 and p65 in WT and STAT3KO macrophages following LPS stimulation. Interestingly, nuclear translocation of NF-κB subunits p65 in STAT3KO increased after incubation with LPS for 9 h (Figure 3b). We further validated this finding by investigating whether the ΙκBα, an upstream protein of NF-κB, might be influenced at later stages of LPS stimulation in STAT3KO cells, we performed immunoblotting after LPS stimulation (0–24 h) and studied the protein expression profiles. Consistent with our nuclear extract results, there were significant differences between WT and STAT3KO macrophages in whole cell lysates (Figure 3c). NF-κB signaling proteins were reduced after 24 h LPS stimulation with different levels between WT and STAT3KO macrophages. These results indicate that STAT3 can regulate *iNOS* gene expression and NF-κB signaling from early to late time points of LPS stimulation.

### 2.4. Effects of STAT3 on NO Production, Invasion, and Colony Formation

**Figure 4:**
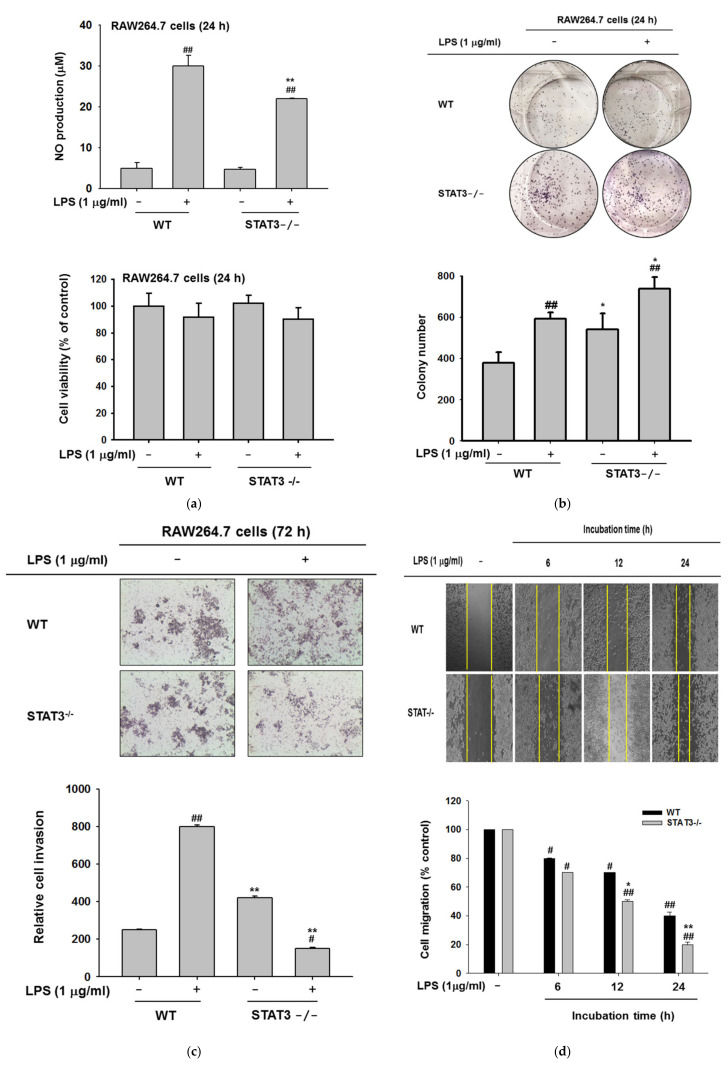
STAT3 deletion impacts NO production, macrophage colony formation, invasion, and cell migration. (**a**) Wild type and STAT3^-/-^ RAW264.7 cells were incubated with LPS (1 μg/mL) for 24 h, and NO production was determined by the Griess assay and cell viability was measured using the MTT assay. (**b**) Wild type and STAT3^-/-^ cells were subjected to a clonogenic assay. Colonies were allowed to form for nine days and cells were left untreated or incubated with LPS (1 μg/mL) for 24 h. Representative images of colonies are shown. Colonies were observed microscopically, and quantification of the number of colonies is shown. (**c**) Wild type and STAT3^-/-^ RAW264.7 cells (5 × 10^4^), either untreated or incubated with LPS (1 μg/mL) were subjected to a transwell invasion (with Matrigel) assay. Cell invasion was imaged under a microscope. (**d**) Cell migration assay performed in wild type and STAT3^-/-^ RAW264.7 cell under LPS stimulation for 6, 12, and 24 h. Representative images are shown at the indicated time points. ^#^*p* < 0.05 and ^##^ *p* < 0.01 compared to normal group in the same cell type and * *p* < 0.05 and ** *p* < 0.01 compared to the same condition of wild type.

To establish the roles of STAT3 in macrophage responses to LPS exposure, we compared the phenotype of STAT3KO and WT macrophages after incubation with LPS. First, NO production was examined in STAT3KO and WT macrophages against LPS exposure. NO production is one of the key anti-bacterial defense mechanisms deployed by macrophages. Nonspecific cytotoxic and inflammatory responses following infection by pathogens results in upregulation of induced NO production by NO synthase [59]. Consistent with our previous findings (Figure 3a), NO production was significantly reduced in STAT3KO cells following 24 h incubation with LPS (Figure 4a upper panel). Furthermore, no significant reduction in cell viability between WT and STAT3KO cells treated with LPS was observed (Figure 4a bottom panel). The clonogenic assay with and without LPS treatment for 24 h showed that STAT3KO macrophages formed more colonies compared to WT macrophages (Figure 4b). However, the increased pattern of colony formation in STAT3KO cells exhibited similarly in wild type cells (Figure 4b), indicating that STAT3 deletion does not greatly affect colony formation of macrophages. Interestingly, we observed that STAT3 silencing significantly increased the cell invasion capabilities in non-treated conditions with respect to WT, whereas LPS treatment for 72 h significantly impaired the cell invasion in STAT3KO cells (Figure 4c). On the other hand, migration was increased in STAT3KO cells upon LPS stimulation from 6 to 24 h in comparison to WT (Figure 4d), suggesting that deletion of STAT3 in RAW264.7 macrophages has a direct inhibitory effect on the promotion of macrophage invasion stimulated by LPS. The findings indicated that STAT3 modulated diverse biological functions under pathogen-infectious conditions.

## 3. Discussion

**Figure 5:**
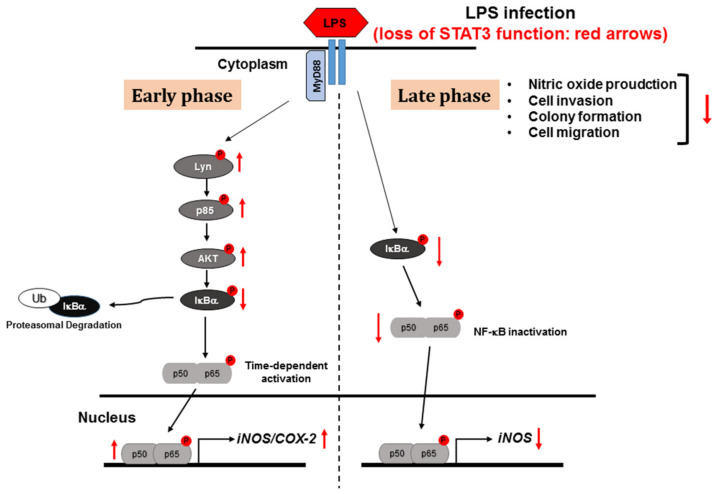
Schematic of inflammatory signaling mechanisms and regulation of STAT3KO macrophages during early and late phases of LPS stimulation.

In the present study, we investigated the roles of STAT3 in the RAW264.7 macrophage cell line after stimulation by LPS. Using a CRISPR-CAS9 approach, we demonstrated that knockout of STAT3 resulted in activation of *iNOS* expression and pro-inflammatory signaling pathways leading to the activation of the upstream membrane protein Lyn tyrosine kinase at early time points. In contrast, at later time points, LPS stimulation attenuated *iNOS* gene expression and NF-κB subunit translocation into the nucleus in STAT3KO cells. The results of the NO production and invasion assays suggested that STAT3 promotes NO production and cell invasion in macrophages. Taken together, we demonstrated that STAT3 co-regulates *iNOS* gene expression and inflammatory signaling pathways according to the duration of LPS stimulation (Figure 5).

STAT3 is an important transcription factor that regulates the expression of numerous transcripts involved in immune response, cell cycle control, and development [60]. Upon stimulation with TLR agonists, growth factors, and cytokines, STAT3 is activated by phosphorylation at T705 and S727. Recent studies have identified that STAT3 inhibition with JSI-124 activates the NF-κΒ signaling pathway at 15 min post-treatment with a consequent increase in pro-inflammatory cytokines [61]. In addition, STAT3 inhibition is known to modify gene expression and immune responses. In particular, inhibition of STAT3 signaling during LPS stimulation led to time-dependent increases in TNF-α, IL-1β, IL-6, and IFN-β [62]. The crosstalk between NF-κB and STAT3 have been studied at various levels—for example, activation of STAT3 by NF-κB regulated factors like IL-6 and COX-2 [63,64]. In the current study, we found that blockade of STAT3 signaling induced greater early activation of the NF-κΒ subunits p50 and p65 into the nucleus upon LPS stimulation in comparison to WT macrophages (Figure 2b) and upregulated the gene expression and protein levels of *iNOS* and *COX-2* but not cytokines at early time points (0–60 min) (Figure 2a); interestingly, at late phase (24 h), the expression of inflammatory genes such as *TNF-α* and *IL-6* but not *iNOS* and *COX-2* was increased in STAT3KO cell compared to WT cells (Figure 3a).

In the classical inflammatory cascade, the activation of NF-κΒ by TNF-α or LPS involves phosphorylation and activation of the IKK complex (IκBα and IKK) [65] upstream of IκΒα. The IKK complex is responsible for phosphorylation and degradation of IκΒα to free NF-κB subunits in the nucleus [61]. However, our results revealed that IKKα/β was downregulated in cells lacking STAT3, although Akt/PI3K were greatly activated in STAT3KO macrophages (Figure 2c). It has been shown that IκBα can be degraded by an IKK-independent pathway during one hour of TNF stimulation or in mouse embryo fibroblast (MEFs) lacking IKK [66,67]. In addition, PI3K/Akt and NF-κB signaling pathway are known to be dependent on each other [68]. Moreover, NF-κB, STAT3 and PI3K signaling together play a critical role in cell resistance and survival in B-cells and neoplasms [69]. NF-κB and STAT3 have also been reported to physically interact in various cell types [70]. Our results show that regulation and activation of NF-κB signaling by STAT3 appeared to be independent of IKK activation at early time points.

NF-κB signaling is an attractive target for therapeutic intervention for inflammation [71]. Studies have revealed that targeting NF-κB signaling could provide a new approach for treating inflammatory diseases [72,73]. Therefore, we evaluated weather blocking NF-κB would prevent STAT3KO-induced *iNOS* expression in early time points. Indeed, administration of NF-κB inhibitor BAY11-7082 in STAT3KO macrophages inhibited phosphorylation of NF-κB subunit p65 at 30 and 60 min upon LPS stimulation in comparison to WT counterparts (Figure 2f,g). Notably, BAY11-7082 administration also downregulated *iNOS* expression in STAT3KO macrophages in comparison to WT, suggesting that dual inhibition of STAT and NF-κB signaling could be an attractive target for inflammatory disease. 

Our results show that Lyn was activated in STAT3KO macrophages with and without LPS exposure for 1 h (Figure 2d), indicating that Lyn seems to be a key molecule in mediating basal and early activation of STAT3KO cells. Lyn is a member of the Src family of protein tyrosine kinases, mainly expressed in hematopoietic cells. The activation of Lyn induces phosphorylation of tyrosine residues in immunoreceptor tyrosine-based activation motifs (ITAM) of the receptor proteins, which triggers additional signaling events to increase the formation of signaling complex [74]. These responses are found to manage osteoclast differentiation, insulin signaling pathway, pulmonary barrier integrity, liver regeneration, and proliferation of malignant melanoma cells [75,76]. This enzyme is also known to participate in inducing various inflammatory and allergic diseases via direct or indirect activation of NF-κB [77,78,79]. Lyn phosphorylates various proteins including Syk tyrosine kinase (at 317 residue) [80], PI3K/AKT [81], and TRAF-6/TAK-1 protein complex [55]. CD117, CDK1, PTPRC, and PLCγ2 are also representative binding proteins to Lyn [82]. Functional links between Lyn and STAT3 were also reported previously. Thus, Lyn deletion results in STAT3 activation [83], and Lyn is required for STAT3 phosphorylation in mast cells [84]. During B-cell antigen receptor (BCR) engagement, STAT3 was also found to be activated by Lyn, but not by JAK [85]. Several reports concluded that STAT3 activity is modulated by Lyn, but there is less information regarding the regulation of Lyn activity by STAT3. In addition, suppression of Lyn by PP2 and STS and PI3K by LY294002 was also found to reduce upregulation of *iNOS* and *COX-2* in STATKO cells treated with LPS for 1 h (Figure 2h,i), implying that Lyn/PI3K might play significant role in regulation of NF-κB-dependent gene expression under STAT3KO conditions at earlier time points. These results strongly suggest that interplay between Lyn and PI3K could be involved in to early time points of inflammatory signaling as reported previously [81].

The role of Akt, a downstream protein activated by PI3K [17], in regulation of *iNOS* and *COX-2* expression was studied in primary macrophages with inhibitors of PI3K and Akt. In this study, it was observed that PI3K inhibitors are able to inhibit *COX-2* expression in mouse peritoneal macrophages and NO production [57,86]. Moreover, NF-κB is also reported to be activated via activation of Akt [87]. Furthermore, Src, Syk, and Lyn tyrosine kinases are also known to be involved in NF-κB activation by LPS in RAW264.7 macrophages. In our study, we found that STAT3KO macrophages display strikingly upregulated levels of phosphorylated Akt and Lyn and increased mRNA levels of *iNOS* and *COX-2* in early time points of LPS stimulation (Figure 2). Relevant role of Akt in regulation of *iNOS* and *COX-2* mRNA in STAT3KO macrophages was also elucidated using pharmacological inhibitors which are known to inhibit Akt activity in macrophages [57,88,89]. Treatment with BN82002 [57] and LY294002 [90] inhibited the mRNA expression of *iNOS* and *COX-2* in STATKO cells stimulated with LPS for 1 h (Figure 2h). This result suggests that AKT together with Lyn and PI3K might be functionally involved in controlling gene expression of *iNOS* and *COX-2* during STAT3 depletion conditions.

It is evident that NO might have different roles in early or late phases of inflammation. Inhibition of NO can exacerbate disease processes depending on the stage of the disease, and NO produced by *iNOS* influences cerebral ischemic injury in early stages [91,92]. Moreover, NO produced by constitutive NOS (cNOS) is involved in the development of inflammation in early phases and NO from *iNOS* maintains inflammation at later stages [93]. Consistent with the findings of other reports, we demonstrated that STAT3 differentially governs *iNOS* expression according to the duration of LPS stimulation. We also validated the mRNA expression of SOCS3 which is known as a negative regulator of JAK/STAT3 [94]. Although SOCS3 is the known target of STAT3 in IL-10-stimulated conditions [95]; however, we found no significant difference in SOCS3 expression (Figure 3a). In contrast, IL10 expression was induced only in WT cells but not STAT3KO cells, suggesting that STAT3 is not involved in regulation of IL-10 expression upon LPS stimulation [96].

Activated macrophages invade through various matrices to reach injured or inflamed sites [97,98], but STAT3KO macrophages did not progress to become more invasive than WT macrophages under LPS stimulation (Figure 4c). It has also been reported that STAT3 ablation reduced tumor growth, and the tumor cells did not progress to become more invasive than STAT3-expressing tumors [99]. In addition, immune cells proliferate rapidly within inflamed sites [100], so we confirmed the proliferation of the macrophage cell lines using a clonogenic assay (Figure 4b). We found STAT3KO colony formation increased in comparison to WT cells in the absence of LPS. With regard to nitric oxide production (Figure 4a), STAT3 appeared to modulate inflammatory responses in distinct ways.

In conclusion, we demonstrated the diverse time-dependent roles of STAT3 in LPS-stimulated inflammatory signaling. In macrophages lacking STAT3, expression of *iNOS* was altered, and the NF-κB signaling pathway was oppositely regulated. Particular proteins have different roles in cellular responses in accordance with cellular location or phase of the cell cycle [101,102], and our results supported the distinct roles of STAT3 in inflammation are dependent of the duration of LPS stimulation. Overall, we ascertained that STAT3 dynamically influences TLR4-mediated inflammatory molecules.

## 4. Materials and Methods

### 4.1. Materials

LPS (*E. coli* 0111:B4), BN82002, and BAY-11-7082 were purchased from Sigma Chemical Co. (St. Louis, MO). LY294002 was purchased from EMD Millipore (Billerica, MA, USA). PP2 (4-amino-5-(4-chlorophenyl)-7-(t-butyl)pyrazolo[3,4-d]pyrimidine) and staurosporine (STS) were purchased from Merck (Germany). Fetal bovine serum (FBS) and Roswell Park Memorial Institute (RPMI) 1640 were obtained from Gibco (Grand Island, NY). RAW264.7 cells, a BALB/c-derived murine macrophage cell line (ATCC No. TIB-71). Antibodies to phospho-specific and total protein of p50, p65, IκBα, IKKα/β Catalog No.#2694) ERK, JNK, Akt, STAT3, Syk, Src, Lyn, p85, β-actin, and lamin A/C were obtained from Cell Signaling (Beverly, MA, USA).

### 4.2. STAT3 Knockout Cell Preparation

A CRISPR-CAS9 approach was used for STAT3 deletion. Guide RNA was designed according to a CRISPR designing tool (dna20.com). A STAT3-targeted sequence (F-AAGGGCCGGTCCGGGTGCAT; R-ATGCACCCGGACCGGCCCTT) was chosen. The sgRNA was cloned within a lentiCRISPRv2 vector (plasmid ID #52961; Addgene, Cambridge, MA, USA). Transfection and puromycin (1–3 μg/mL) selection were completed using a previously established protocol [38]. To prepare control wild type (WT) cell line, we transfected TrueGuide™ sgRNA negative control, non-targeting 1 (Invitrogen, Cat. No.: A35526, ThermoFisher Scientific, Waltham, MA, USA).

### 4.3. Cell Culture

RAW264.7 and STAT3KO macrophages were cultured in RPMI 1640 supplemented with 10% heat inactivated FBS and antibiotics (penicillin and streptomycin) and were maintained at 37 °C in 5% CO_2_. For each experiment RAW264.7 and STAT3 knockout cells were harvested with a cell scraper.

### 4.4. Migration Assay

Cell migration was assessed by wound-healing assays. Briefly, confluent RAW264.7 and STAT3 knockout cells (1.2 × 10^6^ cells/mL) cultured with or without LPS stimulation were plated on six-well plates ad were wounded by manual scratching with a 200 μL pipette tip. Subsequently, cells were washed with PBS and incubated at 37 °C in complete media. At the indicated time points, phase-contrast images at specific wound sites were obtained.

### 4.5. Transwell Migration and Invasion Assays

The ability of cells to invade through Matrigel-coated filters was determined using 24-well plates with 8-mm pore polycarbonate membranes either uncoated (for migration) or pre-coated with Matrigel Basement Membrane Matrix (for invasion; BD Biosciences). RAW264.7 and STAT3 knockout cells were seeded at a density of 5 × 10^4^ cells/well in 100 μL RPMI containing 5% FBS in the upper compartment of transwell plates. To determine the invasive effect, cells were incubated with and without LPS in the upper compartment of transwells, while complete medium with 10% FBS (1000 μL) served as the chemoattractant in the bottom chamber. After incubation for 72 h at 37 °C in 5% CO_2_, the cells that had not penetrated the filter were completely removed with a cotton swab, and the cells that had migrated to the lower surface of the filter were fixed, stained, and counted in five randomly selected microscopic fields (100×) per filter.

### 4.6. Nitric Oxide Assay

RAW264.7 and STAT3KO cells (1 × 10^6^ cells/mL) were seeded on 96-well plate and incubated for 18 h and then treated with LPS (1 μg/mL) for 24 h. After 24 h, 100 μL of the cell supernatant and 100 μL of Griess reagent were mixed and absorbance was measured at 540 nm using spectrophotometer.

### 4.7. Cell Viability Assay

After overnight pre-incubation of RAW264.7 and STAT3 knockout cells (1 × 10^6^ cells/mL), cells were incubated with or without LPS for 24 h. The cytotoxicity of each inhibitor was evaluated using the MTT assay as previously reported [103]. Three hours prior to culture termination, 10 μL of MTT solution (10 μg/mL in phosphate-buffered saline [PBS], pH 7.4) was added to the cultures, after which the cells were continuously cultured until termination of the experiment. The incubation was halted by the addition of 15% sodium dodecyl sulfate to each well to solubilize the formazan [17] and the absorbance was measured using a Spectramax 250 microplate reader at 570 nm.

### 4.8. mRNA Isolation and Real-Time PCR

RAW264.7 and STAT3 knockout (1 × 10^6^ cells/mL) were incubated with LPS (1 μg/mL) for various durations, and total RNA was isolated using TRIzol Reagent (Gibco BRL, Waltham, MA, USA) according to the manufacturer instructions. RNA samples were then quantified, and one microgram of total RNA was reverse transcribed with M-MuLv Reverse Transcriptase (New England Biolabs, Ipswich, MA, USA) according to the manufacturer instructions. mRNA expression was than studied by real-time PCR using SYBR-premix Ex Taq according to the manufacturer instructions (Takara Bio Inc, Shiga, Japan). Reactions were performed in real-time using a thermal cycler (Bio-Rad, Hercules, CA, USA). Results represents the mean values obtained from three independent experiments. Full description of the primer sequence used are in Table 1. Relative mRNA was calculated by normalizing the values of indicated genes compared to that of housekeeping gene β-actin. Difference was calculated for each treatment using normalized values.

### 4.9. Preparation of Total and Nuclear Cell Lysate

RAW264.7 and STAT3 knockout cells (1 × 10^6^ cells/mL) were incubated with or without LPS for 0–60 min. The cells were then harvested and washed with ice-cold PBS. The pellet was then lysed with ice-cold lysis buffer containing (20 mM Tris-HCl, pH 7.4, 2 mM EDTA, 2 mM EGTA, 50 mM glycerol phosphate, 1 mM DTT, 2 μg/mL aprotinin, 2 μg/mL leupeptin, 1 μg/mL pepstatin, 50 mM PMSF, 1 mM benzamide, 2% Triton X-100, 10% glycerol, 0.1 mM sodium vanadate, 1.6 mM pervanadate, and 20 mM NaF). Total cell lysates were centrifuged at 12,000 rpm for 5 min at 4 °C and stored at −20 °C until use. Nuclear cell lysates were prepared as previously described [16]. Cells were harvested and washed with 1 × PBS at 12,000 rpm for 5 min. The supernatant was discarded, and the cell pellets were lysed in 400 μL of ice-cold lysis buffer (10 mM HEPES PH 7.8, 10 mM KCl, 2 mM MgCl_2_, 0.1 mM EDTA, 1 mM DTT, 0.1 mM PMSF, 2 μg/mL leupeptin, and 2 μg/mL aprotinin). 25 μL of 10% NP-40 was added to the lysates, and the lysates were vigorously vortexed. The lysates were then subjected to centrifugation at 14,000 rpm for 0.5 min at 4 °C, and the supernatant was removed. Pellets were repeatedly washed with 400 μL of lysis buffer and then 50 μL of extraction buffer (containing 10 mM HEPES pH 7.8, 50 mM KCl, 400 mM NaCl, 0.1 mM EDTA, 1 mM DTT, 0.1 mM PMSF, 2 μg/mL leupeptin, 2 μg/mL aprotinin, and 10% glycerol). The solution was stored at 4 °C for 20 min and centrifuged at 14,000 rpm at 4 °C for 5 min. The supernatant was transferred to fresh tubes and stored at −80 °C until required for testing.

### 4.10. Immunoblotting Analysis

Cell lysates were electrophoresed on SDS-polyacrylamide gels (30% acrylamide, Tris-base, 10% SDS, 10% APS, TEMED) using running buffer (Tris-base, glycine, 10% SDS). The proteins were transferred by electroblotting to a polyvinylidene difuoride membrane using transfer buffer (Tris-base, glycine, 10% SDS, methanol). Membranes were blocked using 3% bovine serum albumin (BSA) in TBST at room temperature for 1 h, followed by incubation with specific primary antibodies for 1 h at room temperature or overnight at 4 °C. After primary antibody incubation, the membranes were washed with 0.1% TBST (Trisbase, NaCl, 0.1% Tween 20, pH 7.6) three times for 10 min each, incubated with HRP-linked secondary antibodies with 3% BSA for 1 h at room temperature, and washed with 0.1% TBST three times for 10 min each. Phosphorylated and total forms of p65, p50, Src, p85, AKT, IKKα/β, IκBα, Lyn, p85, β-actin, iNOS, COX-2, and lamin A/C were visualized using an ECL reagent [104]. For calculating relative intensity of all immunoblotting results, the blots were analyzed using ImageJ analysis tool software developed by NIH. Higher resolution immunoblot was imported in ImageJ and were converted to 8-bit format. The individual lanes were selected using rectangular section tool. The program then plots rectangular around all the other bands and quantifies the intensity of each bands. The intensity of each of the phospho-proteins was normalized with the intensity of loading controls β-actin or their total proteins.

### 4.11. Statistical Analysis

Data are presented as mean ± standard deviation calculated from three replicates. Comparisons between multiple time points were analyzed by using an analysis of variance and Scheffé’s post-hoc test, as well as the Mann–Whitney test, and significance was analyzed by the log-rank test. In all the tests, *p* values of less than 0.05 were considered statistically significant.

## Figures and Tables

**Table 1:** Primer sequence used in quantitative RT-PCR.

Gene	Direction	Primer Sequence
*iNOS*	Forward	AGGAGGAGAGAGATCCGATTTAG
	Reverse	CTACTGAGACAGGGAAGTCTGA
*TNF-α*	Forward	CTGGAGGACAGAGAAGAAATGG
	Reverse	AGATTGCCACAGAATCCTGG
*IL-6*	Forward	ACCCTTCCAGATGGCAATATC
	Reverse	CTACTCTATGCTGGGCAGTTT
*COX-2*	Forward	CACTACATCCTGACCCACTT
	Reverse	ATGCTCCTGCTTGAGTATGT
*IL-10*	Forward	TCCTGCCATCACCTGAAATATG
	Reverse	CTTTCTCCTCCTCTGCTTTCTC
*SOCS-3*	Forward	TGTGAAGAGGCAGTAGCATTTA
	Reverse	CAGATCAACAGATGAGCCATCT
*IRF-3*	Forward	CAACCAACAAGTGATATTCTCCATG
	Reverse	CAGGCCATCAGCAACAACAT
*GAPDH*	Forward	CAATGAATACGGCTACAGCA
	Reverse	AGGGAGATGCTCAGTGTTGG

## Abbreviations

ERK	Extracellular signal-regulated kinase
iNOS	Inducible nitric oxide synthase
cNOS	Constitutive nitric oxide synthase
BCR	B-cell receptor
JNK	c-Jun N-terminal kinase
KO	Knockout
IBD	Inflammatory bowel disease
LPS	Lipopolysaccharide
MAPK	Mitogen-activated protein kinase
NF-κB	Nuclear factor-κB
NO	Nitric oxide
PCR	Polymerase chain reaction
STAT	Signal transducers and activators of transcription
TLR	Toll-like receptor
TNF	Tumor necrosis factor
JAK	Janus kinase
CRISPR	Clustered regulatory interspaced short palindromic repeats
